# Life history parameters of *Bemisia tabaci* MED (Hemiptera: Aleyrodidae) in the present and future climate of central Europe, predicted by physically realistic climatic chamber simulation

**DOI:** 10.1093/ee/nvad023

**Published:** 2023-03-18

**Authors:** Milan Milenovic, Michael Eickermann, Jürgen Junk, Carmelo Rapisarda

**Affiliations:** Environmental Research and Innovation Department (ERIN), Luxembourg Institute of Science and Technology (LIST), 41, Rue du Brill, L-4422 Belvaux, Luxembourg; Dipartimento di Agricoltura, Alimentazione e Ambiente (Di3A), Università degli Studi di Catania, Via Santa Sofia 100, 95123 Catania, Italy; Environmental Research and Innovation Department (ERIN), Luxembourg Institute of Science and Technology (LIST), 41, Rue du Brill, L-4422 Belvaux, Luxembourg; Environmental Research and Innovation Department (ERIN), Luxembourg Institute of Science and Technology (LIST), 41, Rue du Brill, L-4422 Belvaux, Luxembourg; Dipartimento di Agricoltura, Alimentazione e Ambiente (Di3A), Università degli Studi di Catania, Via Santa Sofia 100, 95123 Catania, Italy

**Keywords:** whitefly, Aleyrodidae, development, climate change, Luxembourg

## Abstract

Whiteflies of the *Bemisia tabaci* species complex are among the most damaging insect pests in agriculture worldwide, causing damage by feeding on crop plants and by vectoring plant viruses. The species complex consists of over 35 cryptic species that differ in many aspects of their biology including the optimal environment, geographic distribution, and host range. Global warming and associated climate change resulting from human activities is expected to contribute to biological invasions. *Bemisia tabaci* species show fast adaptability to changes in agroecosystems and have a long record of biological invasions. Climate change driven increase in *B. tabaci* importance in agricultural systems of Europe has been predicted, but so far not experimentally tested. The present study evaluates the development of *B. tabaci* MED (=Mediterranean) in a climatic chamber simulation of the future climate in Luxembourg, chosen as a representative region for the Central Europe. Future climate predictions for the period 2061–2070 were derived from a multimodel ensemble of physically consistent regional climatic models. Results show a 40% shorter development time of this important pest in future climatic conditions, with an increase in fecundity by a third, and insignificant difference in mortality. Accelerated development, combined with its already established year-round presence in European greenhouses and predicted northward expansion of outdoor tomato production in Europe, means faster population build-up at the beginning of the outdoor cropping season with the potential of reaching economic importance. Benefits of simulating hourly diurnal cycle of physically consistent meteorological variables versus previous experiments are discussed.

## Introduction

*Bemisia tabaci* species complex is a highly invasive and devastating sap-sucking pest group feeding on of hundreds of plant species including ornamentals and many crops such as tomato, pepper, eggplant, cucumber, cotton, sweet pepper, and tobacco (Byrne and Bellows Jr 1991). Whitefly feeding causes direct damage in form of chlorosis, stunted growth, and even wilting (Byrne and Bellows Jr 1991). More importantly, feeding is associated with the transmission of numerous plant viruses (Byrne and Bellows Jr 1991, Brown 1994, Rajinimala et al. 2005, Jacobson et al. 2018). While the species complex has global presence, the species within differ in their geographic distribution, host preferences, and apparent aggressiveness and invasion potentials (Brown 2009). Among the numerous species of this complex, *B. tabaci* MED (=Mediterranean) is characterized by its higher level of resistance to many classes of insecticides and a wider host range (Iida et al. 2009, Ran et al. 2018, Wang et al. 2018, Xie et al. 2018, Horowitz et al. 2020, Park et al. 2021). Furthermore, it is more aggressive as witnessed in China, where MED introduction led to displacement of MEAM1 (=Middle East Asia Minor 1), that was already established in the area (Xie et al. 2018). In Europe, two most dominant *B. tabaci* species, MEAM1 and MED, present in protected crops across all Europe, while restricted to warmer areas of Mediterranean in the open field (Bertin et al. 2018). While the two species can coexist in certain areas, superior insecticide resistance of MED often makes it dominant in highly intensive agricultural systems (Bertin et al. 2018). In the EU, non-European populations of *B. tabaci* are considered as a A1 quarantine pest (Annex II A), and in the United Kingdom, Sweden, and Ireland, it is classified as protective zone quarantine pest (Annex III) (The European Commission 2019, 2021). With its presence in protected cropping systems, there is a danger of easy spread of *B. tabaci* MED to the open field if, or rather when, climatic conditions become suitable enough (Nauen et al. 2002).

With ongoing climate change and predicted warming of European climate (Allan et al. 2021), accurate assessment of this risk is crucial for the development of sustainable climate change adaptation, and plant protection strategies. Development of *B. tabaci* (sensu lato) has been shown to be primarily determined by temperature, with relative humidity at both extremes causing reduced survival (Byrne and Bellows Jr 1991, Aregbesola et al. 2019). Investigations into the effects of elevated CO_2_ levels indicated little to no effect on whitefly development (Curnutte et al. 2014). Whiteflies compensate for the lower nutritional value of the phloem sap of the plants grown under elevated CO_2_ by longer phloem ingestion feeding phase (Peñalver-Cruz et al. 2020).

Numerous studies have focused on creating detailed models of whitefly’s temperature-dependent development (Butler Jr et al. 1983, Bethke et al. 1991, Powell and Bellows Jr 1992, Drost et al. 1998, Muñiz and Nombela 2001, Musa and Ren 2005, Bonato et al. 2007, Aregbesola et al. 2020, Chandi et al. 2021). The input data for these models are experimentally determined developmental parameters at a range of constant temperature/humidity values. The outcome is therefore a very precise characterization of the development at given constant conditions. These models however do not consider the effect of the diurnal cycle of environmental parameters on the insect development. By extension, these models might not be representative of the development under natural conditions. Traditionally, the insect development models are then combined with climate data to produce a model of insect performance in different climates, which further approximates and simplifies the complex interactions between the insect and the environment (Gilioli et al. 2014, Ramos et al. 2018, Bradshaw et al. 2019). No studies so far have tried to model or experimentally simulate jointly the effect of changing temperature, relative humidity, and CO_2_ concentrations on whiteflies.

The present study assesses the *B. tabaci* MED development on tomato under projected Central European future climate conditions in a more realistic way by simulating the climate in a climate chamber based on hourly data of relevant atmospheric variables. Physical climate simulation allows whitefly development to be measured rather than simulated in silico. Therefore, we used present time series from historical observation data, and future time series from physically consistent, regionally downscaled multi-model climate projections to control the climate chamber experiments.

## Materials and Methods

### Plant Material

Tomato plants of the cultivar “Moneymaker”, grown from seed (Kiepenkerl, Bruno Nebelung GmbH, Everswinkel, Germany) were used for whitefly rearing and climate simulation experiments. Plants used for maintaining laboratory colony of *B. tabaci* were grown in an insect-proof cage (50 × 50 × 50 cm) at 22°C, 50% RH, and photoperiod of 12:12 (L:D) h under Valoya C65 (Valoya Ltd., Helsinki, Finland) lights with NS12 spectrum. Soil moisture content was maintained at 70–80% field capacity, and once plants reached the 5th leaf stage, they were fertilized weekly by irrigating with 200 ml of Peters Professional Allrounder (Dublin, OH, United States) water-soluble fertilizer at the concentration of 2 g/liter. Plants used in the climate simulation experiment were grown from seed at both environmental conditions described below, with the same irrigation and fertilization regime as the one reported above.

### Insect Material

The previously characterized laboratory whitefly colony of *B. tabaci* MED was used as a source of insect material (Milenovic et al. 2022). The population harbors primary endosymbiont *Portiera* (group P1), and two secondary endosymbionts, *Rickettsia* (group R1, with scattered phenotype), and *Arsenophonus* (group similar to A2c, with bacteriocyte confined phenotype) (Milenovic et al. 2022).

### Climate Simulation

To assess the development of whiteflies under present and future environmental conditions, a climate change experiment was carried out in Bronson Incrementum 1400 and 1500 (Bronson Climate b.v., Zaltbommel, The Netherlands) climatic chambers equipped with Valoya NS12 luminaries, set to deliver 480 µmol/m^2^ s photosynthetic photon flux density (PPFD) at 20 cm distance. The CO_2_ concentration was maintained at 410 ppm and 700 ppm for the present and future conditions, respectively.

Diurnal courses of temperature and relative humidity for present condition were obtained from an automatic weather station (AWS) of the official agrometeorological network of Luxembourg (Obercorn, 49° 51ʹ N, 5° 90ʹ E, 378 m above mean sea level). Luxembourg was selected as an example of Central European country where *B. tabaci* MED is currently absent but could be introduced and potentially established in the future. Long-term hourly values (2006–2015) of air temperatures and relative humidity for that AWS were retrieved from the data archive operated by Administration des services techniques de l’agriculture (ASTA). A long-term (2006–2015) diurnal course of mean July air temperatures based on hourly values was calculated. Based on the daily mean air temperature of that long-term daily course, a representative day was selected from the original data set of the AWS (19.07.2007).

Results of regional climate change projections for Luxembourg were used for the climate change impact assessment (Goergen et al. 2013, Junk et al. 2019). The future climate time series to drive the climatic chamber simulations were derived from regional climate projections, taken from the Coordinated Regional Climate Downscaling Experiment project (CORDEX, http://www.cordex.org). However, climate projections of regional climate models still have biases when compared to observational data (Kotlarski et al. 2014) which precludes their direct usage in climate impact studies based on absolute values. A common approach is the use of bias-adjustment methods to reduce those biases. In the current study, CORDEX data was corrected using a state-of-the-art quantile mapping method for the time series of air temperature and humidity. Besides the correction of the mean of the distribution, also the width and the shape of the distribution were corrected. The method is described in detail by Themeßl et al. (2011) and Wilcke et al. (2013). A multi-model ensemble of regional climate models (eight ensemble members) forced with the RCP8.5 emission scenarios (Representative Concentration Pathway) delivered physical consistent future meteorological variables and CO_2_ levels for the time-span 2061–2070. An hourly multi-model mean of the daily long-term July air temperatures and humidity projections was calculated and the differences were added to each hour of the original hourly time series obtained from the AWS at Obercorn.

**Fig. 1.**
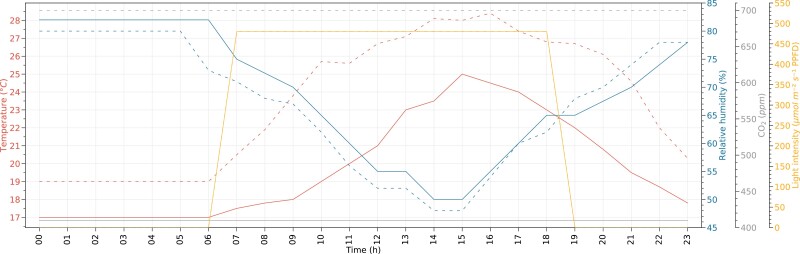
Daily courses of air temperature, relative humidity, CO_2_ concentration, and light intensity used to drive climate chambers for present and future conditions. Solid and dashed lines represent the data for present and future conditions, respectively.

Daily courses of meteorological parameters used to program the climate chambers for current and future experimental conditions are presented in Fig. 1 and in the Table S1 of the Supplementary Material.

### Whitefly Development

**Fig. 2.**
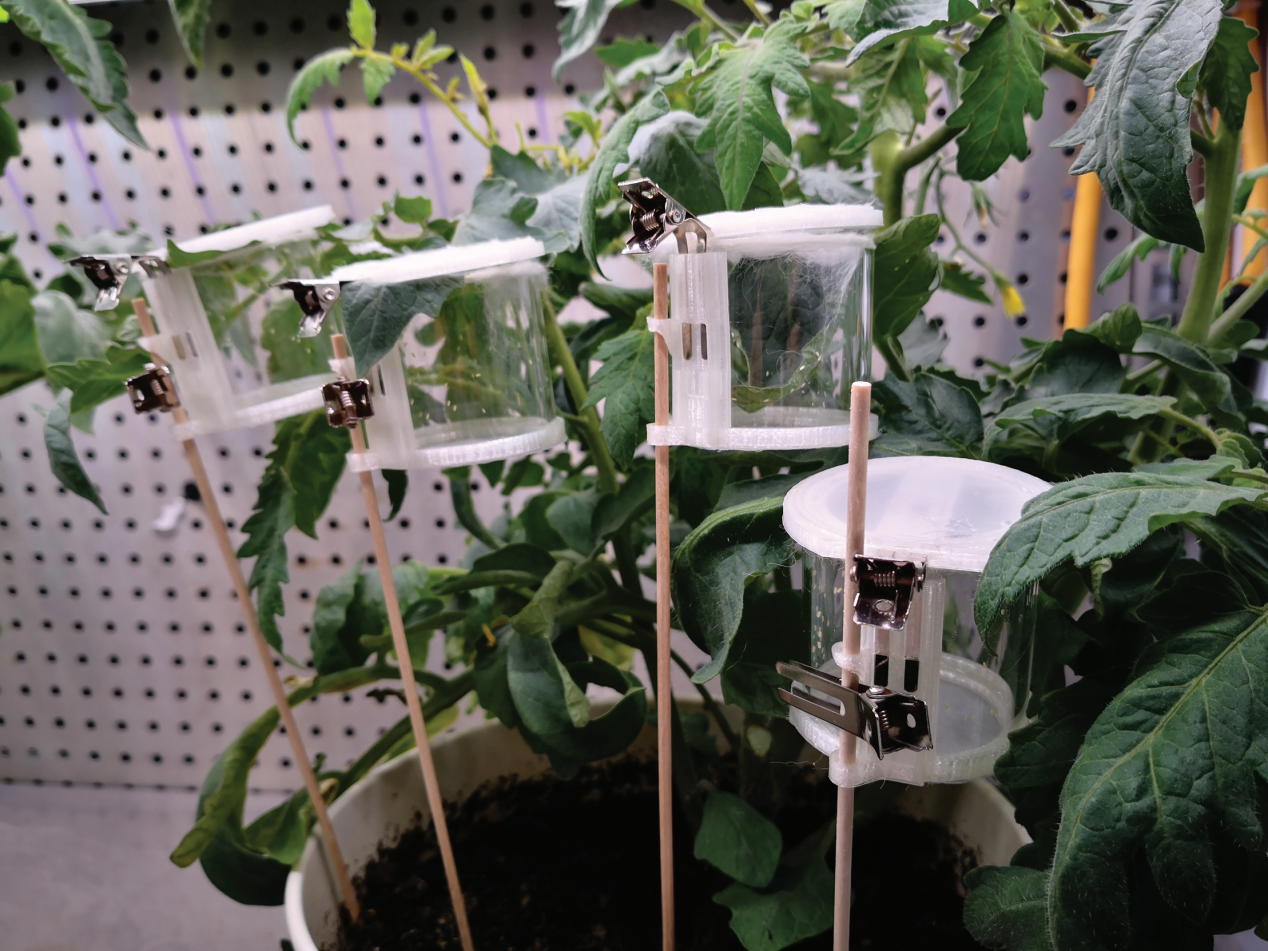
Tomato plant with four clip cages demonstrating the clip cage design and experimental setup. Cage glass cylinder diameter and length are 46 mm and 40 mm, respectively.

Tomato plants were grown from seed in the simulated present and future environmental conditions. Each of the two climatic chambers contained two insect-proof cages (50 × 50 × 50 cm, W:D:H) with three plants per cage. Plants were first sown in a container (3 × 3 × 6 cm) and transplanted to 5-liter pots at the one leaf stage. When plants reached the 10th leaf stage, 250 unsexed *B. tabaci* adults were introduced per plant (e.g., 750 adults per cage). After 10 days introduced adults were removed and 100 eggs (50 per cage) spread across all plants were selected for monitoring of whitefly development. Each day (with the exception of weekends) the number of individuals per development stage (egg, 1st–4th instar nymphs, eclosed pupa cases) deriving from the marked eggs was recorded. Before the first adults have eclosed, leaves hosting individuals under study were placed in a clip cage (Fig. 2) to allow monitoring longevity of the eclosing adults. When half of the monitored nymphs have eclosed, 24 additional, freshly hatched, male–female pairs were collected from other, nonmonitored leaves for fecundity monitoring. Of those, four male–female pairs were placed inside a clip cage (Fig. 2), one cage per plant for the total of six cages, representing six replicates. The number of oviposited eggs was monitored as long as the adults were alive.

### Data Analysis

Raw counts of individuals in each stage were summed with counts of individuals in all later stages. This way counts per each stage are inclusive of those individuals that might have already progressed to a later stage. As time needed to reach any given development stage follows unimodal distribution, an S-shaped curve can be fitted and the time to 50% of individuals reaching a particular stage can be calculated. In other words, a dose–response model can be applied where “dose” is the time, and response is the number of individuals reaching a particular development stage. Curve fitting and parameter estimates were done using “drc” package v3.0-1 in R Studio v1.2.5001 with R v.4.1.1. Best model was selected using “mselect” function of the “drc” package testing from the following models: LL.5, LL.4 LL.3, LL.2, W1.3, W1.4, W2.4, Quan, Cubic, and Lin. According to the “mselect” output presented in Supplementary Table S2, a five-parameter log-logistic model (LL.5) was selected and fitted to the data. The model summary is available in Supplementary Table S3. ED (effective dose) function was used to estimate time it takes for a certain percent of individuals to reach each developmental stage. Function “compParm” of the same package was used to perform statistical comparison of ED50 for each developmental stage between two climatic conditions.

## Results

**Fig. 3.**
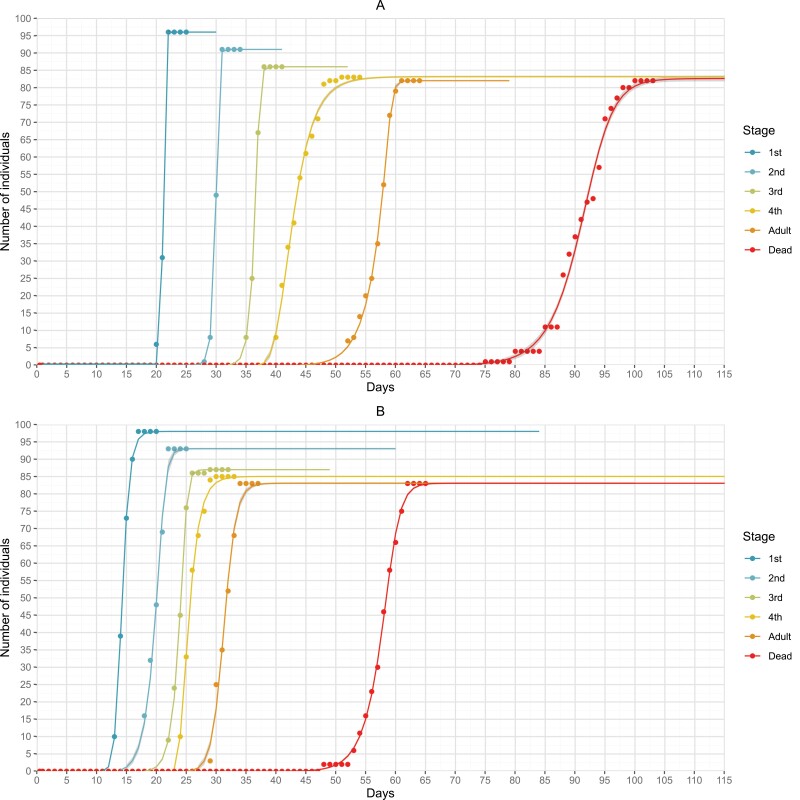
Raw daily counts of *B. tabaci* MED individuals per each developmental stage with fitted dose–response curves for present (A) and future (B) climatic conditions.

**Fig. 4.**
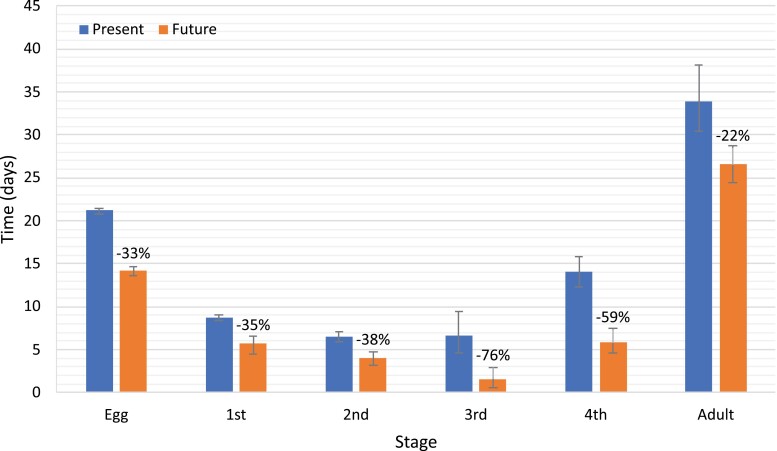
Estimated time to 50% of *B. tabaci* individuals (ED50) reaching the next developmental stage in present and future climatic conditions expressed as development stage duration. Percentage above the “Future” bars represents the change compared to the “Present” bar of the same stage. Error bars represent lower and upper quantiles (ED25 and ED75).

The development of *B. tabaci* MED is significantly accelerated under projected future climatic conditions. In present conditions, 50% of whiteflies reached the first instar stage 21.2 days after oviposition, the second after 29.9 days, the third after 36.5 days, the fourth after 43.2, followed by adult eclosion after 57.3 days (Fig. 3). The adult longevity was 33.9 days in present conditions (Fig. 4). In future conditions, first, second, third, and fourth stage were reached after 14.2, 19.9, 23.9, and 25.5 days, respectively (Fig. 3). Adults eclosed after 31.3 days and had a 26.5 days longevity (Figs. 3 and 4). The differences in the development times between present and future conditions were statistically significant (*P* < 0.01) for all developmental stages. Faster development resulted from the shorter duration of each developmental stage as presented in Fig. 4 and Supplementary Table S4. Statistical parameters including *P*-values are available in Supplementary Table S5. Fecundity of females was also on average higher under future climate conditions, with 85.5 egg per female, compared to 63.7 days per female under present climate conditions (Table 1). Mortality was very similar in present and future climate conditions both in terms of cumulative mortality from egg to adult, as well as for each developmental stage (Table 2).

**Table 1.** Fecundity of *B. tabaci* MED in present and future climate conditions across six clip cage replicates

Eggs/female								
Clip cage replicate #	1	2	3	4	5	6	Mean	Std. Err.
Present	49	55	68	53	59	98	63.7	7.36
Future	103	68	64	85	76	117	85.5	8.48

**Table 2.** *B. tabaci* MED per-stage and cumulative mortality for present and future climatic conditions

	Eggs	1st	2nd	3rd	4th
Present					
Dead individuals	4	5	5	3	1
Mortality (per stage)	4.0%	5.0%	5.0%	3.0%	1.0%
Mortality (cumulative)	4.0%	8.8%	13.4%	16.0%	16.8%
Future					
Dead individuals	2	5	5	2	2
Mortality (per stage)	2.0%	5.0%	5.0%	2.0%	2.0%
Mortality (cumulative)	2.0%	6.9%	11.6%	13.3%	15.1%

Per-stage mortality presents a percentage of individuals of a particular stage that never progressed to the next developmental stage. Cumulative mortality is number of individuals at each stage in relation to the total number of oviposited eggs at the start of the experiment.

## Discussion

The present study is the first comprehensive climate chamber simulation of whitefly development under physically consistent daily variation of air temperature, relative humidity, and CO_2_ concentrations for present and future climate conditions. The simulation reveals a 40% shorter time needed for egg to adult development of *B. tabaci* MED in the future climate (2061–2070, RCP8.5 scenario) compared to the present climate (2006–2015). This acceleration of development will increase the impact of *B. tabaci* MED on the agricultural economy of Central Europe, by building up to significant population levels also in the field during the cropping season. Besides a faster development, a 34% increase in fecundity, without significant change in mortality, will further contribute to faster population increase. Although a study with higher number of individuals under observation would be needed to evaluate the differences in mortality in finer detail, our data shows that the eventual difference is unlikely to counteract increased fecundity.

The year-round establishment of *B. tabaci* MED in the open fields of Central Europe is still unlikely due to the limiting low winter temperatures which interrupt its lifecycle, as shown by life table experiments and reflected in global future distribution models for this pest (Butler Jr et al. 1983, Drost et al. 1998, Muñiz and Nombela 2001, Gilioli et al. 2014, Chandi et al. 2021). However, the year-round establishment is not necessary for *B. tabaci* MED to become an economically important pest of open-field crops in Central Europe since it is already present in the nearby greenhouses, ready to expand as soon as the climatic conditions allow, and able to reach high population levels in only a few generations and a very short time during the spring-summer cropping season.

Climate change will have an effect not only on whiteflies, but also on the distribution of their host plants. Ramos et al. (2018) projected that open-field tomato production will also move and expand further north in Europe and that suitability for *B. tabaci* tightly follows the suitability of tomato in such a way that, by the time open-field production is possible, the risk of *B. tabaci* infestations will already be very significant.

The absence of other studies focused on *B. tabaci* (*s.l.*) performance under controlled conditions with daily courses of air temperature and relative humidity makes it difficult to directly compare development times across the studies. Therefore, we are limited to comparing our results to the studies performed at a constant temperature close to the mean daily temperature for present (19.8°C) and future (23.4°C) conditions from this study. The development times observed in this study are generally slower than those previously reported in the literature at constant temperature for *B. tabaci* MED. Development in the present condition is slightly slower than reported by Bonato et al. (2007) at the constant temperature of 17°C, which is the lowest the authors evaluated. In the same study, authors report time of 39.6 days at 21°C, and 25.6 days at 25°C. When linearly interpolated to the mean temperature in the future conditions of the present study, the egg-adult development is 32.6 days, which is within 2 days from the egg-adult time observed in the present study. On the other hand, results from Muñiz and Nombela (2001) tell a different story. At the lowest tested temperature of 17°C, *B. tabaci* MED had egg-adult development in just 42.7 days, and 24.4 days at 23°C, which is 14.4 and 9.7 days faster than in the present study, respectively. Similar variability of measured developmental parameters is present in other studies (Butler Jr et al. 1983, Drost et al. 1998, Chandi et al. 2021). The observed nonlinearity between constant conditions and realistic daily variation of environmental parameters points out the limits of extrapolating these results to the conditions in nature.

Further potential factors of uncertainty are different host plants, and the unknown composition of the secondary whitefly endosymbionts, both of which are known modifiers of whitefly biology in a context-dependent manner (Powell and Bellows Jr 1992, Drost et al. 1998, Musa and Ren 2005, Milenovic et al. 2021). Regardless of the source of uncertainty, the sheer variability in reported developmental times at the same conditions indicates that obtaining a de facto measurement of *B. tabaci* life parameters is not trivial. Uncontrolled environmental variables, unreported nuances in experimental design, and unknown factors can render the absolute results of insect development incomparable, and by extension almost unverifiable with other studies (Koricheva et al. 1998, Couret and Benedict 2014).

The accelerated development of *B. tabaci* MED under future conditions is dominantly driven by air temperature. Relative humidity was less than 10% different at any given point between the two conditions, with the mean differing by only 1.7%. The effect of elevated CO_2_ alone on whitefly development was out of the scope of the present study. However, previous studies have demonstrated no direct or indirect effect of CO_2_ on whitefly life (Curnutte et al. 2014, Peñalver-Cruz et al. 2020). The present study focused on the impact of future climate and therefore evaluated the impact of joint change of three environmental variables. However, to fully understand the nature of the observed response, future studies employing different experimental designs are needed. Such studies could aim to quantify the effects of diurnal cycle versus constant conditions, and the individual impact of each environmental variable.

Finally, the abovementioned studies that varied only the temperature produced a similar magnitude of change in the development times as the change observed between present and future simulations presented here. The overall accelerated development results from faster development of each life stage. A difference in relative shortening of development time between stages was observed. The duration of the egg, 1st nymphal, and 2nd nymphal stages shortened by around 35%, while the 3rd and 4th nymphal stages shortened by 76% and 59%, respectively (Fig. 4). Adult longevity was reduced by 22%. These differences could partially be explained by the higher overall variability in the stage duration of the latter three stages as shown by the standard error bars (Fig. 4). Further studies are needed to understand the nature of this response.

Summing up, we observed faster development, higher fecundity, and unchanged survival rates point toward higher pest importance of *B. tabaci* MED in Central Europe under future climatic conditions. However, further studies focused on multitrophic interactions are needed, not only to determine if increased activity of natural enemies could counteract the spread of this increasingly important pest (Muñiz and Nombela 2001, Bonato et al. 2007), but also for exploring the different possible behavioral scenarios of this insect species, in future climatic conditions, on different host plants and in populations characterized by different composition of secondary endosymbionts.

## Supplementary Material

nvad023_suppl_Supplementary_Tables
